# Vaccination in the Light of the Royal British Commission

**Published:** 1898-11

**Authors:** M. R. Leverson

**Affiliations:** Fort Hamilton, L. I., N. Y.


### VACCINATION IN THE LIGHT OF THE ROYAL BRITISH COMMISSION.

M. R. Leverson, M. D., Fort Hamilton, L. I., N. Y.

(Session of June 18th, 1890. Continued from Oct. No., page 440.)

Q. 9,964 describes a vaccinal disaster at a place called Motte aux Bois, near Hazebrouck, not far from Lille. From the *Bulletin de L'Academie de Médicine*, No. 37, Dr. Decouvelaere, of Hazebrouck, on the 14th of August, 1889, informed the academy that he had been called to attend a number of children vaccinated thirteen days previously, July 31st, 1889. Out of thirty-eight children vaccinated, thirty-seven presented lesions to which at that time M. Decouvelaere did not attribute a syphilitic character, but seven days later he made known to the academy new facts which had dissipated all his doubts, and which seemed to him to show the truly syphilitic nature of the accidents observed. These variations in diagnosis are unavoidable. On August 25th there were forty-three victims. "The accidents broke out on the eighth or tenth day after the vaccination. On all the children the vaccine had been inoculated by three punctures on one arm only." The vaccine vesicles seem to have appeared on the second or third day. "They were very early the seat of violent imflammation; they rapidly increased in volume, and, with very few exceptions the three vesicles at the end of eight or ten days were charged in the case of each subject to suppurating wounds, the evil appearance of which quickly aroused the parents of the vaccinated children." M. Decouvelaere says: "The least ill exhibit three hermatic* ulcerations the size of a fifty-centime piece. The base of them is of a grayish color, the edges hard, elevated, regular and surrounded with an inflamed aureole more or less extended. Others worse attacked presented larger ulcerations suppurating freely, the edges serrated and irregular, with surrounding inflammation

* Qy. Hermetic?

deeper and considerably more extended; œdema of all the limbs. Others, the most ill, confluent ulcerations forming one wound on the outer region of the arm. Abundant suppurative and considerable œdema. In some cases the erythema produced by contact with the discharge seemed covered with a false skin. In one child the ulcerations had run together and attained the dimensions of a five-franc piece." On August 25th M. Hervieux reports: "Their surface—(*i. e.*, those not cicatrized) was generally smooth pimpled, of a bright red color, like that of a blister, sometimes uneven, grayish, and of unfavorable aspect. The edges which seemed depressed and as if full when the ulcers progressed towards cicatrization, were on the contrary elevated and breaking out in little spots in the case of those whose vaccination wounds had not yet yielded to treatment." There was a great *abundance*, and in some cases a *fetid* suppuration. On a certain number of children pressure of the ulceration disclosed the existence of *an indurated circle* like a ring or belt of leather, but more often, at least at the time M. Hervieux made his examination, the existence of the circle seemed fleshy (or sticky), as if œdematous. In two or three cases a thick, yellowish scab was formed, covering the subjacent ulcer and enclosing it like the glass of a watch. [Compare any good description of the Hunterian chancre.—M. R. L.]

Q. 9,965. In spite of all the above, M. Hervieux goes on to express his faith in vaccination, and

Q. 9,966. Mr. Fournier gives a summary of the cases, and suggests that these disasters are cases of *ulcerous vaccination*. But a woman whose fingers were probably infected with vaccinal pus showed at the corner of the lower eyelid after having rubbed her eye *an ulceration resembling a chancre*. [And after all, if the patient is made seriously ill, the constitution invaded and of necessity the vitality lowered, what does it matter to him by what *name* the nosologist may choose to designate his malady? —M. R. L.]

Q. 9,967. The next point is the cutaneous symptoms following vaccination at Elberfeld. In *Le Progrès Medical*, No. 44, Vol. VIII, November 3d, 1888, p. 323, it is said: "Dr. Pourquier read a paper on 'The cutaneous symptoms consequent upon animal vaccination, the causes, the influence on the culture of the vaccine matter, and the means for preventing them.' Similar symptoms have been observed in Germany by Protze of Elberfeld. In Germany there were eight hundred infant patients who had all been vaccinated from the same two calves. Ulcerations developed on the surface of the pustules, then there appeared some vesicles resembling the phlyctaena of the pemphigus ; these burst and were replaced by yellowish crusts." "Dr. Pourquier states as the result of researches made on bacteriological culture that these epidemics are due to the presence of a micro-organism." [See, on the germ theory, HOMŒOPATHIC PHYSICIAN, Vol. XVIII, pp. 263–269 ; also *Origines Épidemiques*, by Dr. Boucher, of Saint Servain, France. Nancy, 1896.—M. R. L.]

"Professor Bruardel refers to an epidemic consequent upon vaccination, where a large number of children exhibited impetiginous symptoms ; sixteen vaccinated children died in twenty-four hours. The heifer was thoroughly healthy—*i. e., before* they began to poison it ; it was the third collection of vaccine matter which was toxic.

Q. 9,969. Another epidemic, taken from the (London) *Lancet* of December 13th, 1866 ; it is headed, "Syphilis extensively propagated by vaccination in France." At Morbihan, in a western department of France, severe syphilitic symptoms developed in more than thirty children from vaccination.

Q. 9,970. Puts in a letter from Dr. Robert Brudenel Carter to the *Lancet.* He advocates vaccination, but recommends its postponement till the child is nine or twelve months old, and other precautions, and says : "I am quite aware that there is now a sort of common consent among medical writers to gloss over the evils that may be attendant upon vaccination for the sake of its great and manifest benefits. In this cause I cannot concur," etc. Also quotes from a leading article in the *Medical Times and Gazette*, February 1st, 1878, referring to the discussion on Mr. Hutchinson's cases, then recently brought before the Medico-Chirurgical Society, in which the practice of keeping cases of disasters from vaccination secret was referred to. Mr.

Tebb reports conversation with a professor of surgery at the University of Christiania, who said: "Yes, I have known cases of syphilis; we have had some, but you know we say little about them."

Q. 9,971. Quotes Dr. Ernest Hart's work, *The Truth About Vaccination; an Examination and Refutation of the Assertions of the Anti-Vaccinators.* At page 29, speaking of vaccino-syphilitic inoculation, he says: "During the twenty years in which there has been systematic inspection of public vaccination in England, some millions of vaccinations have been performed, but in no single instance have the government inspectors of vaccination been able, after the most rigid inquiry, to find one single case of syphilis after vaccination."

Q. 9,972. The book was published in 1880.

Q. 9,973–80. Dr. Hutchinson, Professor Michael Foster, and the chairman hasten to show that Dr. Hart's statement refers to England, but (Q. 9,980) in the evidence before the select committee in 1871, besides four hundred and fifty cases of vaccinal syphilis in France, numerous medical witnesses report syphilitic cases. They are found in Sir John Simon's book: "There was an inquiry made by means of circulars by Sir John Simon, in the year 1856, and here are the answers. I have had a summary made out. The report is contained in a blue book, entitled *General Board of Health: Papers Relating to the History and Practice of Vaccination.* Presented to both Houses of Parliament by command of Her Majesty in 1857. These papers contain five hundred and thirty-nine replies from medical authorities." Dr. Schieferdecher "gives an analysis of these replies, and divides those which contain reasons unfavorable to vaccination under four heads, as follows: I. It directly endangers life. II. It nurses and develops latent diseases. III. Children frequently do not thrive so well after as before vaccination, especially during teething, change of teeth and puberty. IV. It introduces new diseases into the system of the vaccinated patient."

Q. 9,982. "Forty-seven of the replies admit that other diseases are introduced into the system by vaccination; twenty admit the inoculation of syphilis, and twenty-two admit explicitly that health is 'disadvantageously affected by the operation.'"

At the session of the 25th of June, 1890, Mr. Tebb reverts to Mr. Hart's pamphlet published in 1880, with the approval of the Local Government Board, in which it is asserted that "in no single instance have the government inspectors of vaccination been able, after the most rigid inquiry, to find one single case of syphilis after vaccination," and at Q. 9,997, refers to Mr. Jonathan Hutchinson's well-known cases of invaccinated syphilis, which he had published in the *Med.-Chir. Trans*, 1871 and 1873, and which were well-known to the medical department at the very time they "approved" Mr. Hart's untruthful pamphlet.

Reverting to the session of the 18th of June, Mr. Tebb says:

Q. 9,983. "In the same blue book is a report of the Royal College of Physicians, published in the year 1807, and mention is there made of a report of the Royal College of Surgeons. But while the report of the College of Physicians is printed as part of the case for vaccination, the report of the Royal College of Surgeons is omitted." Mr. Tebb then speaks of the trouble he had had in procuring a copy of the original document which is entitled, "Report of the Royal College of Surgeons of London on Vaccination, with an appendix containing the opinions of the Royal Colleges of Physicians of Edinburgh and Dublin, and of the Royal Colleges of Surgeons of London, of Dublin, and of Edinburgh. Ordered to be printed 8th July, 1807."

Q. 9,984. Describes the formal proceedings of the Royal College of Surgeons of London, and a summary of the results of their investigations, as follows: Number of persons reported by 426 correspondents, 164,381; number of cases of smallpox which had followed vaccination, 56; bad consequences, eruptions, 66 cases; inflammation of the arm, 24 cases, of which three proved fatal.

Q. 9,985–6. (By Mr. Meadows White.) The report of the Royal College of Surgeons was embodied in the report of the Royal College of Physicians, but is omitted in the volume prepared for Parliament by Sir John Simon.

Q. 9,988–90. (By Professor Michael Foster.) The other reports which are embodied in the Report of the Royal College of Physicians of London, are given by Sir John Simon; he only omits the report of the Royal College of Surgeons.*

Q. 9,991. Mr. Tebb next proceeds to consider the connection between vaccination and leprosy. He hoped and used every effort to induce the gentlemen who had advised him upon the subject and who were best informed and most competent to do so to come forward to testify, and "it is only because I have failed in this regard † and in view of its great importance, that he brings the matter before the Commission."

He proposes "to deal with the facts under the following heads:

---

* Dr. Hart's work, *The Truth about Vaccination*, quoted by Mr. Tebb (Q. 9,971) went through a second, or revised edition, which was published under the approbation of the Local Government Board, yet the statements quoted by Mr. Tebb are contained in it, notwithstanding Mr. Simon's report of 1857, which Mr. Hart mentions with absurd eulogy in his preface as having been largely used by him. Mr. Jonathan Hutchinson's cases of vaccinal syphilis were also well known to the entire profession and were testified to by him before the Select Committee in 1871. At Q. 9,984–90, Mr. Tebb, as we have seen, laid bare the falsification of public records by Sir John Simon in that very report of 1857 which Dr. Hart calls "a masterpiece of medical essay writing." Pretending to report to Parliament the entire report of the Royal College of Physicians of 1807, he did present, as parts of it, the reports of the Royal Colleges of Surgeons of Edinburgh and Dublin, and suppressed the report of the College of Surgeons of England which also formed part of the report of the Royal College of Physicians of England, and is *the only one of all* the reports which was made *after investigation*.

Such instances of direct fraud and falsehood are not exceptional, and have been paralleled in the United States. One of the saddest features of the history of vaccination is the unveracity it has developed on the part of a very large number of its official defenders, and the degradation of the medical profession consequent upon the establishment of a State Medical Church.—M. R. L.

† It appears from Mr. Tebb's work, *The Recrudescence of Leprosy* (Swan Sonnenschein & Co., London), a volume of over 400 pages, dedicated to the Royal Commission, that the information he secured was obtained in most cases from officials high in the service of the British Government. The fact that they could not be induced to testify before the Commission, furnishes some slight indication of the sort of terrorism exercised by officialism in England. Every physician in the United States who has had the courage, after investigating vaccination, to come forward publicly as its opponent, has had to suffer something of the like persecution.—M. R. L.

1. The serious increase of leprosy. 2. Evidence that leprosy may be propagated by inoculation. 3. Evidence as to its diffusion by vaccination."

As all these matters are fully discussed by Mr. Tebb in his work, *The Recrudescence of Leprosy*, and as unlike the British Blue Books, this work is readily accessible *and very readable*, the Editor presents only the more important portions of Mr. Tebb's testimony on this subject.

With regard to the first; the sources of his information as to the Virgin Islands, the Leeward and Windward Islands, and to British Guiana, are: governors, medical practitioners, superintendents of leper hospitals, prison chaplains, and editors of newspapers.

In some islands, such as Jamaica, St. Kitts, and Trinidad, there are leper communities which are gradually increasing.

Q. 9,993. The leper asylum at Mucurapo, Port of Spain, Tinidad, visited in February, 1889, contains 180 patients, admirably cared for by the French Dominican sisters. Dr. Rake, the medical superintendent, in his report to the Surgeon-General for 1887, says: "The new infirmary at the asylum was opened in August last, and was quickly filled." A new one was to be built at once. At the asylums at Mahaica and Gorchun there were about 500 lepers, and according to the Berbice paper, where the asylum is located, the accommodation was insufficient, and lepers in the worst stages of the malady were to be seen daily in the streets and byways.

As to British Guiana, Dr. C. F. Castor, in his report to the Surgeon-General of the Colony for 1887, says that leprosy is spreading very considerably. Mr. Tebb reports conversations with various medical officials in Guiana, with Governor Haynes Smith and others, to the same effect.

In a leading article in the *St. Christopher Gazette* (St. Kitts), May 17th, 1881, entitled "The most pressing question in the Colony," the writer quotes Dr. Boon's quarterly report, which he says "clearly and forcibly showed the government the enormous increase in our leper population during the last six years," and the writer attributes such increase to vaccination.

In the *Journal of the Royal Agricultural and Commercial Society*, of Demerara, 1889, Dr. John D. Hillis, for ten years medical superintendent of the leper asylum, Mahaica, British Guiana, gives emphatic testimony to the alarming increase of leprosy in that colony.

Governor Wm. Robinson, of Trinidad, writing to the Secretary of State from Government House, May 9th, 1889, says, that after fifteen years' residence in the West Indies he fully corroborates Dr. Rake's statement that leprosy is on the increase. Mr. Tebb's next point is, is leprosy inoculable? It is not necessary to discuss this question to a medical audience, unless, indeed, some one of the numerous readers of THE HOMŒOPATHIC PHYSICIAN should have the hardihood to deny it.

One medical man did once have the courage to deny to me that Sydenham ever said, "if no mistake be made by physician and nurse, small-pox is the lightest and safest of all diseases," but he was not a reader of THE HOMŒOPATHIC PHYSICIAN. There is also considerable evidence tending to show that, apart from inoculation, leprosy is not contagious. Testimony is then given as to the enormous increase in leprosy in the Sandwich Islands, a fact of the deepest significance to us, now that the annexation of those islands has become an accomplished fact, and Dr. Edward Arning, who came from Hamburg to investigate the causation of the increase of leprosy, in his report to the Board of Health, Honolulu, dated Nov. 16th, 1885, says: "Closely allied to the inoculation question is the subject of vaccination. * * * There have been, if my information is correct, unquestionably new centres of leprosy developed after vaccination was practised."

In a summary of reports furnished by foreign governments to his Hawaiian Majesty's authorities as to the prevalence of leprosy in India and other countries, and the measures adopted for the social and medical treatment of persons afflicted with the disease, are the following extracts from the *Medical and Surgical Journal*, April, 1880: "Vaccination was also inquired into. Alarmed by an invasion of small-pox in 1853, a general vaccination of the whole population was ordered, and physicians being at that time very few on the islands, non-professionals aided in the work. It is charged * * * that not only syphilis, but also leprosy was greatly increased." In his handbook on the *Diagnosis of Skin Diseases*, 1880, pp. 284, 285, Dr. Siveing writes: "Leprosy has within the last thirty years been imported and spread rapidly among the natives of certain islands (the Sandwich Islands) *where it was before quite unknown.*"

Dr. Robert Pringle, Surgeon-Major late Sanitary Department Her Majesty's Bengal Army, says in a letter to the *London Times*, June 12th, 1889, "Knowing what I do about the infection of small-pox, I am amply justified from a careful study of small-pox inoculation and vaccination during the whole of my thirty years' Indian service, in stating that unless prompt and stringent measures are taken in Bombay, leprous inoculation will become far more possible and hence probable than it may appear at present."

Mr. Tebb next quotes a lecture by Sir Wm. Moore, late Surgeon-General Bombay Staff and Head of the Medical Department, Western India, given at King's College Hospital (No. 38 of the Hospital Association pamphlets, pp. 2, 3), in which a number of cases of the inoculation of leprosy are given. He also quotes the report of Dr. Heidemstam, chief medical officer for Cyprus sent by the High Commissioner Sir Henry Bulwer to Lord Knutsford, and presented to Parliament, March, 1890, also showing that leprosy is a strictly inoculable disease.

